# Trafficking of Kainate Receptors

**DOI:** 10.3390/membranes4030565

**Published:** 2014-08-20

**Authors:** Steffen Pahl, Daniel Tapken, Simon C. Haering, Michael Hollmann

**Affiliations:** 1 Department of Biochemistry I, Ruhr University Bochum, Universitätsstr. 150, 44780 Bochum Germany; E-Mails: steffen.pahl@rub.de (S.P.); daniel.tapken@rub.de (D.T.); simon.koesters@rub.de (S.C.H.); 2 International Graduate School of Neuroscience, Ruhr University Bochum, Universitätsstr. 150, 44780 Bochum, Germany; 3 Ruhr University Research School, Ruhr University Bochum, Universitätsstr. 150, 44780 Bochum, Germany; 4 Graduate School of Chemistry and Biochemistry, Ruhr University Bochum, Universitätsstr. 150, 44780 Bochum, Germany

**Keywords:** kainatereceptor, trafficking, endocytosis, retention, assembly

## Abstract

Ionotropic glutamate receptors (iGluRs) mediate the vast majority of excitatory neurotransmission in the central nervous system of vertebrates. In the protein family of iGluRs, kainate receptors (KARs) comprise the probably least well understood receptor class. Although KARs act as key players in the regulation of synaptic network activity, many properties and functions of these proteins remain elusive until now. Especially the precise pre-, extra-, and postsynaptic localization of KARs plays a critical role for neuronal function, as an unbalanced localization of KARs would ultimately lead to dysregulated neuronal excitability. Recently, important advances in the understanding of the regulation of surface expression, function, and agonist-dependent endocytosis of KARs have been achieved. Post-translational modifications like PKC-mediated phosphorylation and SUMOylation have been reported to critically influence surface expression and endocytosis, while newly discovered auxiliary proteins were shown to shape the functional properties of KARs.

## 1. Introduction

The protein family of iGluRs comprises three receptor subfamilies whose distinction has historically been based on sequence homologies and pharmacological properties of the proteins. Classified after their most potent agonists, these receptor subclasses have been named *α*-amino-3-hydroxy-5-methylisoxazole-4-propionic acid (AMPA), N-methyl-D-aspartate (NMDA), and kainate (KA) receptors (see [1] for review). All iGluRs share a tetrameric subunit composition as well as a common subunit topology in which the N-terminal domain (NTD) is located extracellularly, succeeded by three transmembrane domains (TMD A through TMD C, or TMD 1 through TMD 3, also known as membrane domains M1, M3, and M4), a re-entrant loop (pore loop or M2) penetrating the membrane from its intracellular side between TMDs A and B, and a cytoplasmic C-terminal domain (CTD) [2,3,4,5]. The ligand-binding domain (LBD) of iGluRs comprises two lobes, formed by the S1 and S2 domains of the subunit. The S1 domain precedes the TMD A, whereas the S2 domain is defined as the region between TMDs B and C [6,7]. Although all of these receptors are ligand-gated ion channels, they serve distinct functions within neurons in particular, and generally within the CNS. The probably most striking and unique functional feature of KA receptors (KARs) is their ability, despite operating in canonical ionotropic signaling, to be involved in non-conventional metabotropic signaling pathways (see [8] and [9] for review). Among the iGluRs, KARs act as key players in the regulation of synaptic network activity (reviewed in [10]) and are widely expressed in the CNS in complex expression patterns [11]. Proper functionality of neuronal networks requires precise fine-tuning of neurotransmission, and misguided localization of the receptors that confer this transmission likely results in severe neuropathologies. Indeed, several studies pointed out that KAR dysfunction or deregulated KAR abundance are implicated in severe diseases of the brain (reviewed in [12]), such as schizophrenia [13], recurrent major depressive disorder [14], and autism [15]. However, KARs comprise the probably least well understood group of iGluRs, and their functional properties, regulation, and modulation remain elusive until now. Nevertheless, important advances in our understanding of the mechanisms involved in the regulation of KAR abundance and function have been achieved over the past years. This review will specifically focus on findings that shed new light on the regulation of ER assembly, retention and forward trafficking of KARs as well as their endocytosis from the plasma membrane and dynamic interaction with auxiliary proteins at the synapse.

## 2. Mechanisms of Assembly and Surface Expression of Kainate Receptors

Based on their affinity for kainate, KAR subunits are classified into low-affinity and high-affinity subunits. KARs can assemble as homomeric or heteromeric tetramers composed of various combinations of the five subunits GluK1 through GluK5. However, the number of possible subunit combinations is limited by the fact that only low-affinity subunits (GluK1 through GluK3) can form functional homomeric receptors, while it is mandatory for the high-affinity subunits GluK4 and GluK5 to co-assemble in a complex together with low-affinity subunits to form functional KARs. On the other hand, the diversity of KARs is increased by post-transcriptional modifications such as RNA editing and alternative splicing (see [16] for review).

### 2.1. Homomeric KARs and Trafficking/Retention Motifs

Endoplasmic reticulum (ER) retention signals are known to be involved in the regulation of ER release of KAR subunits in many cases. Zerangue and colleagues were the first to report a novel sequence acting as an ER retention signal that regulates the surface expression of potassium channels [17]. A stretch of three basic amino acids (RKR) has been recognized as being critical for the observed ER retention, and it has been demonstrated that the RKR motif causes ER retention when fused to proteins otherwise trafficked to the plasma membrane. Similar retention motifs have since been identified in the NMDA receptor subunit GluN1 (RRR) [18,19] and subsequently in the KAR subunits GluK1, GluK3, and GluK5 [20,21,22,23,24].

#### 2.1.1. GluK1

**Figure 1:**
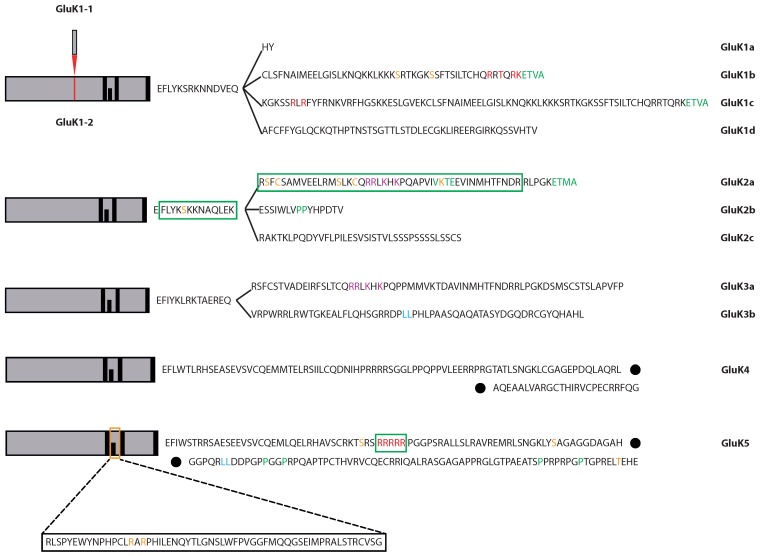
Splice variants of KARs. Splice variants are depicted including reported interaction sites with other proteins, post-translationally modified residues, and trafficking motifs. Color index: red: retention motif; purple: forward trafficking motif; light blue: endocytosis; orange: post-translational modification; green: residues or regions reported to interact with other proteins. Dark bars within schematic drawings of receptors represent either transmembrane domains or the re-entrant loop. References for KAR sequences: [25,26,27,70,71,72,73,74,75,27,70].

GluK1 undergoes alternative splicing within the N-terminal (NTD) and C-terminal domains (CTD). Splicing within the NTD results in two variants GluK1-1 and GluK1-2, while the four CTD splice variants were termed GluK1a, GluK1b, GluK1c, and GluK1d (Figure 1), which is only found in humans and therefore will not further be discussed in this review [25,26,27]. Additionally, the diversity of GluK1 is further increased by the existence of two editing variants resulting from Q/R editing in its pore loop (see [16] for review). GluK1a and GluK1b are present at the plasma membrane, although the majority of subunits remains within the ER, whereas GluK1c expression is robustly confined to the ER [21]. Indeed, it has been shown for GluK1-2b that ER retention at least partly results from a motif consisting of a positively charged arginine (R896) and surrounding amino acids located within the alternatively spliced C-terminal region as well as two basic amino acids (R900 and K901) further downstream [23]. In addition, insertion of a phosphorylation-mimetic mutation (T898E) near the ER retention motif promoted ER exit and surface delivery of GluK1-2b receptors, suggesting that the trafficking of this subunit could possibly be phosphorylation status-dependent [23]. The particularly strong retention of GluK1c instead is related to an RXR motif within the CTD [21] similar to the motifs reported for potassium channels and NMDA receptors [17,18,19].

#### 2.1.2. GluK2

GluK2 diversity is, similarly to GluK1, increased by editing and by alternative splicing within the CTD. GluK2 can undergo editing within the first transmembrane domain (I/V and Y/C editing) and Q/R editing within the pore loop, while alternative splicing happens exclusively at the CTD, resulting in the three variants GluK2a, GluK2b, and GluK2c (Figure 1 or [16] for review). Both GluK2a and GluK2b have been reported to be targeted to the plasma membrane, yet this targeting is distinctively stronger for GluK2a, which is attributed to a stretch of amino acids containing four basic residues (873 RRXKXK 878) that serve as a forward trafficking motif [21]. Comparable results were obtained by Yan and colleagues, who proposed a stretch of seven amino acids (871 CQRRLKH 877) largely overlapping with the aforementioned sequence to be critical for surface expression of GluK2a (Figure 1). The residues C871 and R873 were experimentally shown to be of particular importance, because their mutation causes a major reduction in surface expression levels [28]. Furthermore, C871 is known to be a target site for palmitoylation (Table 1), which was shown to inhibit phosphorylation of GluK2 by PKC when palmitoylated [29], suggesting it to play a role in anchoring the receptor’s CTD at the plasma membrane. Nasu-Nishimura *et al*. extended this work and identified S846 and S868 as phosphorylation sites for PKC in GluK2a (Table 1). GluK2b lacks the second serine residue. Phosphorylation at both sites was shown to reduce ER exit of GluK2 [30]. Furthermore, profilin IIa, an actin-binding protein involved in the regulation of membrane transport of proteins [31], inhibits exocytosis of GluK2b-containing KARs by binding to the CTD of GluK2b via a diproline motif [32,33].

#### 2.1.3. GluK3

GluK3 diversity is increased by alternative CTD splicing resulting in the two splice variants GluK3a and GluK3b (Figure 1). Similar to GluK2, GluK3a is highly expressed at the plasma membrane, while the b isoform is strongly retained in the ER. Surface expression of GluK3a is promoted by the same forward trafficking motif already described to influence the trafficking of GluK2a. However, the RXR motif within the CTD of GluK3b does not appear to be involved in ER retention of the protein [34], unlike what has been reported for GluK1c and GluK5 [21,24]. Indeed, surface expression of GluK3b seems to be regulated by a dileucine motif within the subunit’s CTD, leading to rapid endocytosis and degradation of plasma membrane-expressed GluK3b-containing KARs. In addition, in COS-7 cells a substantial amount of GluK3b has been shown to be immaturely glycosylated, as evidenced by its endoglycosidase H sensitivity. By contrast, GluK3b carrying a mutated dileucine motif was not EndoH-sensitive, suggesting that the dileucine motif may also be involved in ER retention of GluK3b [22].

#### 2.1.4. GluK4 and GluK5

The first study showing that homomeric GluK5 receptors are retained in the ER identifies a stretch of five arginine residues (RRRRR) within the CTD (Figure 1) to be involved in this mechanism [20]. Disruption of this motif results in an increased surface expression of homomeric GluK5 receptors. Furthermore, it has been shown that this poly-arginine ER retention motif interacts with the coatomer protein complex I (COPI) [35], which is thought to function as a mediator of ER-retrieval of misfolded or unassembled proteins from the Golgi back to the ER [36]. A similar arginine-rich motif is also present within the CTD of the related subunit GluK4, suggesting that both KAR subunits are retained at the ER via similar mechanisms, although experiments proving this assumption have not been conducted so far [20]. Subsequently, an additional motif (RXR) in the intracellular loop preceding the second transmembrane domain of GluK5 has been identified to mediate ER retention (see Figure 1). Simultaneous mutation of the previously identified and the new motif resulted in considerably higher surface expression of GluK5 than the single mutation of the CTD motif [24]. However, both studies confirmed that plasma membrane-expressed GluK5 does not form functional homomeric ion channels. In addition, GluK5 carries a dileucine endocytosis motif in its CTD, which is believed to be involved in clathrin-mediated endocytosis and therefore serving as another checkpoint to prevent inappropriate surface expression of homomeric GluK5 [20].

### 2.2. Assembly and Trafficking of Heteromeric KARs

#### 2.2.1. Structural Determinants of Subunit Assembly and ER Exit

The precise mechanisms of KAR subunit assembly still remain largely unresolved. However, Ayalon and colleagues proposed a model [37] in which the NTD mediates the initial subunit association into dimers, while assembly of fully functional receptors into a tetramer is induced by the compatibility of other regions. Such regions to be critical for mediating assembly may be the transmembrane region and the C-terminal part of the S2 domain [37]. Indeed, subsequent work confirmed that KAR assembly involves an initial dimerization step of the subunits’ NTDs, showing that high-affinity NTD interactions facilitate the biosynthesis of functional heteromeric receptors [38]. Nevertheless, it has been shown in truncation experiments that NTD assembly is not mandatory for expression of functional homomeric KARs [39].

Another important constituent in the regulation of KAR surface expression seems to be the ligand binding domain (LBD). Mah *et al*. demonstrated that disruption of ligand binding in GluK2 leads to reduced agonist-induced currents below detectable levels accompanied by an intracellular retention of subunits. As evidenced by electrophysiological experiments, surface expression could be restored by coexpression of the binding site mutants with GluK2 wild type subunits, indicating that the inserted mutation did not affect subunit oligomerization [40]. In addition, it has been shown that heteromeric KARs incorporating mutated GluK5 subunits with reduced agonist affinity are still able to co-assemble with either GluK1 or GluK2. However, the surface expression of these heteromeric receptors is markedly reduced. This suggests that agonist binding and the associated conformational changes are a prerequisite for efficient exit from the ER and serve as a quality control step for forward trafficking of assembled receptors [41]. Additionally, it has recently been demonstrated that agonist binding to only the GluK5 subunit of heteromeric GluK2/GluK5 KARs is necessary and sufficient for trafficking of the receptors to the cell membrane. These findings suggest a distinct role of GluK5 in the regulation of KAR surface expression and might serve as a mechanism that preferentially favors the formation of heteromeric KARs in the presence of high-affinity KAR subunits [42].

In fact, it has additionally been shown that glutamate binding is essential for proper folding of the LBD of GluK2a. Briefly, following agonist-induced channel-activation, KARs rapidly desensitize. In the desensitized state the receptor is closed although the agonist is still bound to the LBD. Following removal of the agonist the receptor recovers from desensitization into the deactivated state and can again be activated by agonists (see [43] for a review on gating and permeation of KARs). It has now been demonstrated that relaxation from the closed, glutamate-bound conformation and the associated conformational changes are necessary for efficient receptor biosynthesis and trafficking [44]. In the same way, inhibition of desensitization by stabilization of the binding domain through the introduction of intermolecular disulfide bonds leads to increased ER retention of GluK2 homomers [45]. In addition, changes in the S2-M3 linker region, which is thought to transduce ligand binding into channel opening, influence the biogenesis of KARs. Mutation of R663 in this region impacts desensitization, attenuates oligomerization, and increases degradation of the subunits, supporting the importance of this region for tetrameric assembly and the existence of a post-assembly trafficking checkpoint [46]. Another mechanism shown to be involved in the regulation of surface expression of heteromeric KARs is RNA editing. Ball *et al*. showed that Q/R editing of GluK2 leads to a reduced plasma membrane expression of receptors, resulting from attenuated oligomerization and decreased ER exit and stability of GluK2(R)-containing KARs [47]. However, how these structural characteristics influence the trafficking of KARs on a mechanistic level has yet to be determined.

#### 2.2.2. Heteromeric Assembly, Trafficking and Endocytosis

Several studies indicate that the assembly of heteromeric KARs is favored over the homomeric assembly of subunits [38,42,48]. Membrane delivery depends on subunit composition of the receptor complex, splice variants and editing status of the subunits [21,23,34,47,49]. Recently, it has been shown that GluK2/GluK5 heteromeric receptors initially assemble as heterodimers, providing a mechanism that ensures a 2:2 stoichiometry and favors heterotetrameric assemblies [38,48]. Still, co-existence of GluK2 homomers and GluK2/GluK5 heteromers has been seen in other studies [49,50], indicating the existence of additional mechanisms to be involved in the regulation of KAR assembly.

Nevertheless, heteromerization seems to be a key determinant in the regulation of KAR surface expression. It was shown that readily plasma membrane-expressed subunits, such as GluK2a and GluK3a, in heteromeric assembly promote the surface expression of otherwise ER-retained subunits or splice variants [21,34,51], probably by sterically shielding the ER retention/endocytic motifs of the normally ER-retained subunits. For example, although GluK5 is able to at least dimerize in the ER, homomeric GluK5 fails to be transported to the plasma membrane, whereas co-expression of GluK2 disrupts the ER retention of GluK5 and allows the resultant heteromeric GluK2/GluK5 complex to escape from the ER [49,52]. Disruption of GluK5 ER retention is at least partly administered by a reduced COPI binding to GluK5 after oligomerization with GluK2, accompanied by an increased association of 14-3-3 proteins [35], which are known to promote forward trafficking of other proteins [53,54,55,56]. Indeed, 14-3-3 proteins have just recently been shown to alter functional properties of GluK2a and GluK2a/GluK5 KARs and associate with them in a PKC-dependent manner [57].

Furthermore, in the hippocampus the amount of plasma membrane-expressed GluK5 critically depends on the presence of GluK1 and GluK2 [58,59], whereas both GluK4 and GluK5 apparently do not co-assemble with GluK3 in the hippocampus *in vivo* [60]. However, the overall GluK5 surface expression in the brain remains unchanged in GluK1-/- mice [24,47], indicating that GluK2 is the major assembly partner of GluK5. Furthermore, at least for GluK2, Q/R editing influences the surface expression of KARs and serves as both a rate-limiting factor in ER exit and a mechanism that favors heteromeric assembly with GluK5 [47].

The subunit composition of KARs is also believed to be involved in regulating their subcellular distribution. Kayadjanian *et al*. showed that GluK1 was confined to proximal dendrites in cultured hippocampal neurons when expressed alone, whereas it was localized at distal dendrites when co-expressed with GluK2 and GluK5. Furthermore, GluK1 expression exhibited a strong overlap with the organization of the microtubule network. In fact, the microtubule motor protein KIF17, which interacts with GluK2 and GluK5 *in vivo*, is required for GluK1 localization to distal dendrites, probably by forming a complex of GluK1 and GluK2 or GluK5 subunits with KIF17 [61]. This study is the first example of a novel mechanism by which the KAR distribution along hippocampal dendrites is determined by the receptor subunit composition.

Furthermore, it has been shown that endocytosis and subsequent degradation of GluK3b-containing KARs are differentially regulated in dendrites and axons of hippocampal neurons, suggesting these two mechanisms to play a prominent role for polarized trafficking of KARs. Endocytosis followed by degradation of GluK3b-containing KARs is driven by a dileucine motif in the CTD of GluK3b (Figure 1). Furthermore, the initial internalization is mediated by clathrin and dynamin2. It the presented study of Huyghe *et al*. it was shown that GluK3a and GluK3b are predominantly expressed in dendrites of neurons. However, preventing endocytosis led to a markedly increased proportion of neurons exhibiting a polarized expression of GluK3b in axons, suggesting that the polarized expression of GluK3b is controlled by endocytic mechanisms [22]. GluK3-containing receptors are thought to be involved in presynaptic facilitation of glutamate release at hippocampal mossy fiber synapses [62], Contradicting this hypothesis, the results of Huyghe *et al*. imply a postsynaptic localization of GluK3. One possible explanation could be that targeting of heteromeric GluK2/GluK3 KARs, which are supposed to represent presynaptic receptors, is differently regulated than targeting of homomeric GluK3. In this case a similar mechanism as described for GluK1 could apply, where GluK1 localization depends on the presence of GluK2 or GluK5 [61].

Recently, an additional mechanism influencing the targeting of neurotransmitter receptors to synapses has been proposed, which is mediated by the BEACH domain protein neurobeachin (Nbea) [63]. Nbea is selectively expressed in neurons and endocrine cells. In neurons, Nbea is recruited to tubulovesicular endomembranes in a GTP*γ*S-stimulated manner [64]. For Nbea KO mice, defects in synapse morphology, enrichment of synaptic molecules, and synaptic transmission have been reported [65,66,67]. It has now been shown that the defects in synaptic transmission in neurons of Nbea KO mice are attributable to a reduced synaptic localization of glutamate and GABAAreceptors, whereas synapse formation and presynaptic function remain unaffected in Nbea KO mice [63]. While the overall expression levels of receptor subunits also remained unchanged, the plasma membrane expression of various AMPA, NMDA, GABA_A_, and KAR subunits was reduced to varying degrees. Reduced plasma membrane expression was accompanied by an increased accumulation of GluA2/3 subunits within the cell body, indicating an unaltered biosynthesis, but deficient trafficking of AMPA receptors in neurons of Nbea KO mice. In addition, it was shown that GluA2/3 subunits exhibited reduced levels of glycosylation, thus implying a compromised transport from the ER to the Golgi apparatus. The EndoH-sensitivity of NMDA, GABA_A-_, and KAR subunits was not altered, indicating these subunits to be arrested late within the Golgi apparatus in Nbea KO neurons [63]. In summary, these results suggest Nbea to be involved in trafficking and postsynaptic targeting of neurotransmitter receptors. Interestingly, SAP-102 (synapse-associated protein 102), a MAGUK (membrane-associated guanylate kinase) which is thought to be involved in the development of excitatory synapses [68], has been shown to bind to the C-terminus of Nbea [69]. These results further suggest that Nbea may be involved in the targeting of neurotransmitter receptors directly to synapses by a mechanism that is probably mediated by the scaffolding protein SAP-102.

## 3. Activity-Dependent Regulation of Synaptic KAR Abundance

### 3.1. PKC-Dependent Phosphorylation of KARs and KAR-Mediated LTD

The first study providing evidence for PKC action on KARs was published by Dildy-Mayfield *et al*. who showed that PKC-mediated phosphorylation of recombinant GluK2 results in decreased kainate-evoked currents [76]. PKC phosphorylates S846 and S868 of GluK2 (1), while palmitoylation at C858 and C871 near the phosphorylation sites negatively influences PKC action on GluK2 [29].

Subsequently, PKC-mediated regulation of KAR abundance and its interplay with scaffolding proteins of KARs was further elucidated. Park *et al*. report that PICK1 interaction with KARs is involved in the expression of EPSC_KA_ LTD in perirhinal cortex layer II/III neurons—a form of LTD mechanistically distinct from EPSC_AMPA_ LTD [77].

Briefly, EPSC_KA_ LTD is induced by activation of inwardly rectifying KARs and requires a postsynaptic rise in the Ca^2+^ concentration that is independent of NMDA receptors and T-type voltage-gated Ca^2+^ channels, suggesting that the KARs directly provide the required Ca^2+^ signal. The influx of Ca^2+^ is thought to trigger further Ca^2+^ release from intracellular stores, which in turn induces LTD. Blockade of mGluR5, PKC, or PICK1 PDZ domain interactions with KARs results in a decrease of EPSC_KA_, which fully occludes EPSC_KA_ LTD [77]. Therefore, Park *et al*. propose a model in which, during basal transmission, EPSC_KA_ is maintained by PICK1-KAR interaction. PICK1 interacts with PKC [78] and GluK1 and GluK2 [79], and therefore localizes PKC to the receptor. Moreover, the function of GluK1-containing KARs is upregulated via a mGluR5-triggered, PKC-dependent mechanism in perirhinal cortical neurons [79,80]. During LTD induction, elevation of intracellular Ca^2+^ is proposed to disrupt the mGluR5-PKC-PICK1-mediated maintenance of KARs, ultimately resulting in decreased EPSC_KA_ [77]. In contrast to EPSC_AMPA_ LTD, the described form of KAR LTD induction is NMDA-receptor independent and requires a precise frequency of stimulation (5 Hz) [77].

In hippocampal neurons, Selak *et al*. identified the scaffolding protein SNAP-25 (synaptosomal-associated protein 25) to be involved in the regulation of EPSC_KA_ LTD [81] by a mechanism distinct from that described above for perirhinal cortex layer II/III neurons [77]. SNAP-25 co-localizes with GluK5 in postsynaptic compartments of mouse hippocampal neurons, interacts with PICK1, GRIP, and GluK5, and appears to be involved in mGluR-mediated, PKC-dependent induction of EPSC_KA_ LTD [81].

Specifically, SNAP-25 regulates GluK5 internalization in an activity-dependent manner. Blocking SNAP-25 activity causes a GluK5-dependent increase in KAR-mediated EPSCs. Furthermore, PKC stimulation triggers EPSC_KA_ LTD without the need of a significant postsynaptic Ca^2+^ rise. Briefly, a model was proposed in which, upon mGluR activation, PICK1 mediates PKC-dependent phosphorylation of GluK5, inducing an association of GluK5 with SNAP-25, and in parallel decreasing GRIP binding affinity to GluK5, ultimately leading to reduced membrane stability of GluK5-containing KARs. However, the precise mechanism how SNAP-25 is involved in regulation of GluK5 internalization still needs to be elucidated [81]. In support of this mechanism, SNAP-25 has recently been shown to be involved in endocytic events at a conventional synapse. In rat hippocampal neurons, SNAP-25 plays a role in both slow, clathrin-dependent endocytosis and exocytotic mechanisms [82].

Quite obviously, the proposed models of EPSC_KA_ LTD differ strikingly in the role PKC activity plays during induction of LTD. While Park *et al*. and Hirbec *et al*. suggest PKC activity to increase the stability of KARs at the membrane [77,79], Selak *et al*. provide a mechanism in which PKC activity leads to destabilization of KARs in the membrane [81]. This apparent discrepancy could result from different behavior of the same proteins in different synapses, or the distinct composition of KARs within the characterized synapses.

Indeed, PKC action seems to exert a bidirectional effect on KARs, apparently depending on the experimental setup and KAR composition. Cho *et al*. show that mGluR5-triggered activation of PKC results in increased EPSCs mediated by GluK1-containing KARs in perirhinal cortex neurons [80]. By contrast, activation of KARs stimulates the phosphorylation of GluK1-2b by PKC at residues S880 and/or S886, resulting in an increased internalization of GluK1-2b. However, the alternative splice variant GluK1-2a does not seem to be phosphorylated by PKC. Furthermore, PKC activity was shown to induce the retrieval of KARs from the membrane in dorsal root ganglia (DG) neurons. This process is counterbalanced by calcineurin, which appears to be involved in the reconstitution of KARs to the membrane [83]. The activity of PKC and calcineurin is regulated by the intracellular Ca^2+^ concentration, with the two proteins possessing different Ca^2+^ affinities [84,85,86,87]. Thus, intracellular Ca^2+^ levels could also play a role in regulating the aforementioned processes involved in EPSC_KA_ LTD.

### 3.2. PKA- and Calcineurin-Mediated Post-Translational Modifications of KARs

While PKC-dependent phosphorylation clearly plays a major role in mechanisms regulating synaptic plasticity, cAMP-dependent protein kinase (PKA) appears to be more related to the direct modulation of functional properties of KARs (Table 1). In 1993 it was reported that phosphorylation of GluK2(R) by PKA at residues S666 and S684 potentiates kainate-evoked currents of recombinant receptors [88,89]. However, in a later study and after revision in 1994 of an initially incorrectly assumed four-transmembrane-domain topology of glutamate receptors [3], it became clear that these residues were in fact located in an extracellular domain. These findings prompted Kornreich *et al*. to reinvestigate the PKA phosphorylation sites of GluK2, which eventually were shown to be S856 and S868 at the CTD of GluK2 [90].

Furthermore, Traynelis *et al*. hypothesized that the phosphatase calcineurin might directly dephosphorylate GluK2, thereby reversing the effects of PKA phosphorylation on GluK2 [91]. In support of this work, Ghetti and Heinemann provided evidence for a modulation of KARs in hippocampal neurons by calcineurin and CaMK [92]. Calcineurin is involved in the regulation of KAR currents in an NMDA receptor- or voltage-sensitive Ca^2+^ channel-dependent manner. Ca^2+^ influx into the cell after stimulation is proposed to induce the activation of calcineurin, resulting in dephosphorylation of KARs and subsequently a transient depression of currents. Recovery of the current amplitude, however, is CaMK-dependent [92]. Later on, Coussen *et al*. confirm the direct interaction of GluK2b with calcineurin and suggest that calcineurin binds to the CTD of GluK2b (Table 2), while it actually dephosphorylates the CTD of GluK2a in heteromeric KARs [32]. Further supporting the model of a calcineurin-dependent dephosphorylation causing depression of KAR currents, Rebola *et al*. report that synaptic NMDA receptors induce postsynaptic short-term depression of KAR EPSCs by activation of calcineurin and subsequent dephosphorylation of GluK2b at hippocampal MF-CA3 synapses *in vivo* [93].

### 3.3. CaMKII-Dependent Regulation of KAR Plasma Membrane Expression

Recently, calmodulin-dependent kinase II (CaMKII) was shown to provide a further mechanism for the regulation of activity-dependent KAR-mediated neurotransmission at hippocampal mossy fiber-CA3 synapses [94]. In this mechanism, repeated pairing of pre- and postsynaptic stimulation, which is known to induce AMPA receptor LTP [95], induces a Ca^2+^- and CaMKII-dependent form of EPSC_KA_ LTD acting on GluK5 [94]. GluK5 is phosphorylated by CaMKII at three residues (S859, S892, and T976; Table 1), resulting in increased lateral mobility of KARs, probably by untrapping GluK5-containing receptors from the PSD [94]. Furthermore, CaMKII activity increases the amount of extrasynaptic KARs in the plasma membrane, whereas the abundance of synaptic receptors is reduced. In conclusion, Carta *et al*. propose a model in which KAR-mediated LTD does not depend on endocytosis, but rather on increased mobility of synaptic KARs that results in KARs leaving synaptic sites [94].

### 3.4. SUMOylation of KARs

Another mechanism involved in the regulation of KAR abundance is SUMOylation. So far, GluK2a and GluK3a/b have been identified as targets for SUMOylation [96,97]. In rat hippocampal neurons GluK2a is a substrate for SUMOylation at residue K886 within the SUMOylation consensus site ΨKXE (Table 1). In cultured hippocampal neurons, application of kainate induces rapid SUMOylation of GluK2a, accompanied by increased endocytosis of KARs. However, SUMOylation levels are not elevated after NMDA application, although activation of NMDA receptors also triggers KAR internalization. These observations indicate that multiple KAR endocytosis pathways exist [96]. Additionally, KAR-evoked EPSCs from CA3 neurons in hippocampal slices are reduced when SUMOylation is experimentally facilitated by infusion of the SUMOylation conjugate SUMO-1 (small ubiquitin-related modifier 1), while facilitation of deSUMOylation by SENP-1 (sentrin-specific protease 1) results in an increased amplitude of EPSCs. The fact that deSUMOylation causes an increase in EPSC_KA_ further suggests that, when SUMOylation — and, as a consequence, endocytosis— of KARs is inhibited, exocytosis and lateral diffusion of KARs into the synapse occur [96], similar to what has already been shown for AMPA receptors [98,99].

Indeed, GluK2 internalization has previously been proposed to underlie activity-dependent endocytic sorting processes [100]. Depending on the endocytic stimulus, KARs undergo differential sorting into recycling or degradation pathways [100]. While low-level stimulation of KARs by kainate leads to recycling of KARs, prolonged stimulation results in endocytosis and degradation. Therefore, González-González propose a bidirectional feedback system that increases KAR abundance in weakly active synapses and degrades them at strongly activated synapses [101]. Transient KAR stimulation by kainate further induces redistribution of GluK2 to synaptic sites via metabotropic pathways requiring PKC and G protein activation and translocation of recycling endosomes into spine heads in a Rab11-dependent manner [101]. Nasu-Nishimura *et al*. extended this work and report that PKC activity on S846 within the CTD of GluK2a mediates endocytosis (see 2.1.2 for an overview of phosphorylation sites of GluK2) [30]. Subsequently, Konopacki *et al*. showed that GluK2 is rapidly phosphorylated upon stimulation of KARs by kainate at S846 and S868, and that phosphorylation of S868 is sufficient for promoting SUMOylation and endocytosis of GluK2. However, residual SUMOylation is also observed in phospho-null mutants, suggesting phosphorylation to enhance SUMOylation, but not being an absolute requirement for it to occur [102].

Recently, Chamberlain *et al*. provided further evidence for phosphorylation and SUMOylation to be important mediators of activity-dependent EPSC_KA_ LTD at rat hippocampal neurons. In the proposed model, phosphorylation of GluK2 S846 by PKC promotes recycling and plasma membrane insertion of KARs in the absence of SUMOylation, while phosphorylation of S868 in conjunction with SUMOylation of K886 results in increased endocytosis of KARs and ultimately leads to expression of EPSC_KA_ LTD. Mechanistically, phosphorylation of S868 is believed to be capable of overriding the function of S846 in endocytosis. Therefore, SUMOylation is supposed to be the molecular switch regulating KAR plasma membrane expression during EPSC_KA_ LTD [103].

Since EPSC_KA_LTD at hippocampal mossy fiber-CA3 synapses requires the destabilization of GluK5-containing KARs associated with SNAP-25 [81], Chamberlain *et al*. further propose that KARs removed from synapses during LTD are composed of GluK2 and GluK5, requiring dual synergistic processes initiated by PKC phosphorylation that lead to binding of PICK1 and SNAP-25 to GluK5 and SUMOylation of GluK2 [103].

**Table 1:** Post-translational modifications of KARs.

Protein	KAR subunit	Target	Reference
**Phosphorylation**			
CaMKII	GluK5	S859; S892; T976	[94]
PKA	GluK2(R)	S856; S868	[88,89,90]
PKC	GluK1-2b	S880; S886	[79]
PKC	GluK2a	S846; S868	[29]
PKC	GluK2b	S846	[29]
**Palmitoylation**			
	GluK2	C858; C871	[29]
**SUMOylation**			
Ubc9/PIAS3	GluK2a	K886	[96]
ND	GluK3a/b	ND	[97]

KAR: Kainate receptor; ND: not determined.

## 4. Proteins Interacting with KARs

KARs interact with a large set of proteins at the plasma membrane (Table 2). These interaction partners include, e.g., proteins of the SAP family and other PDZ or CUB domain-containing proteins, or BTB-Kelch protein family members. The following paragraphs will provide a summary of interactions known to influence the stability of KARs within the plasma membrane and/or shaping the receptor’s function as well as the implications of these interactions for downstream signaling pathways. Interestingly, Coussen *et al*. provided evidence for a co-assembly of the GluK2 splice variants GluK2a and GluK2b together with GluK5 into a triheterotetrameric KAR complex. It was further demonstrated that both splice variants interact with different subsets of proteins, therefore greatly increasing the number of possible interacting proteins for a KAR complex [32].

### 4.1. Proteins of the SAP Family

One of the first proteins demonstrated to establish PDZ (postsynaptic density protein (PSD-95), Drosophila disc large tumor suppressor (Dlg1), and zonula occludens-1 protein (zo-1)) domain-mediated interactions with KARs was PSD-95 (postsynaptic density protein 95, also known as SAP-90 (synapse-associated protein 90)). Garcia *et al*. provided evidence for a direct interaction of PSD-95 and SAP-102 with GluK2 and GluK5 [104]. GluK2 binding to PSD-95 is mediated via the interaction of the GluK2 C-terminal amino acid sequence E-T-M-A with the PDZ1 domain of PSD-95 [104]. Functionally, PSD-95 interaction leads to a faster recovery from desensitization of GluK2 homomeric and GluK2/GluK5 heteromeric KARs [105] Furthermore, PSD-95 interaction promotes clustering of KARs [104]. The interaction of GluK5 with PSD-95 is established via binding of the Src homology 3 (SH3) and guanylate kinase-like (GK) domains of PSD-95 to the CTD of GluK5. While the motif mediating the binding of the GK domain to GluK5 is unknown, SH3 binding is established via a P-X-X-P motif, which is also present in GluK4. However, a direct interaction of GluK4 and PSD-95 has not been shown so far [104]. Subsequently, Hirbec *et al*. showed that PSD-95 also interacts with GluK1b and GluK1c (Table 2), which have a similar sequence at the C-terminus (E-T-V-A) as GluK2 (E-T-M-A) [79].

Apart from consequences for receptor-mediated currents and promotion of KAR clustering, binding of PSD-95 to GluK2 has also implications for downstream signaling pathways. PSD-95 associates with GluK2 and mixed-lineage kinases 2 and 3 (MLK2 and MLK3) in a complex via binding of MLK2/3 to the SH3 domain of PSD-95 [106]. This results in activation of JNK kinases [107,108] and an NMDA receptor-independent form of glutamate-mediated excitotoxicity [109,110,111,112], and is linked to neuronal loss upon ischemic injury [112,113,114].

Additionally, SAP-97 co-immunoprecipitates with GluK2 but not GluK5 [104]. SAP-97 binding to GluK2 is established via its PDZ1 domain [115], similar to the way PSD-95 binds to GluK2 (Table 2). Binding of SAP-97 via its SH3 and GK domains to GluK5, however, is probably inhibited by a mechanism of SAP-97 autoinhibition. Although PSD-95 and SAP-97 are homologous, SAP-97 carries an insertion in the U5 region between its SH3 and GK domains and has an extended N-terminus, which apparently interacts with its own SH3 domain. Furthermore, SAP-97 seems to establish an intramolecular SH3-GK interaction. Both the interaction of the N-terminus with the intramolecular SH3 domain and the established intramolecular SH3-GK domain interaction appear to inhibit GluK5 binding [115]. Interestingly, SAP-97 is expressed presynaptically [116], whereas PSD-95 clusters in putative postsynaptic sites during synaptogenesis [117], suggesting that their differential association with KAR subunits might contribute to the polarized distribution of KARs within neurons.

### 4.2. Other Protein-Protein Interactions Established via PDZ Domains

Apart from proteins of the SAP family, several other PDZ domain proteins interact with KARs (Table 2). Hirbec *et al*. show that the GluK1 isoforms GluK1-2b and GluK1-2c as well as GluK2 bind with their C-termini to the PDZ proteins PICK1 (protein interacting with PRKCA 1), GRIP (glutamate receptor interacting protein 1), and syntenin [79]. GRIP and PICK1 are required for maintaining KAR-mediated synaptic function at mossy fiber-CA3 synapses, and blocking the interaction of either one of these with KARs leads to a decrease in KAR-mediated synaptic transmission. Based on their experimental data, Hirbec *et al*. propose a model in which, similar to AMPA receptors, GRIP anchors KARs to the synapse. In their model, GRIP-binding is stabilized by PICK1-targeted phosphorylation of S880 and/or S886 of GluK1-2b by PKC, because both inhibition of PKC or blocking of PICK1 interaction with the receptor results in a rapid decrease in KAR-mediated currents [79].

In support of this hypothesis, NETO2 interacts with GRIP via a C-terminally located PDZ binding motif and overexpression of NETO2 increases the amount of GRIP associated with GluK2, indicating that GRIP plays an important role in stabilizing GluK2 receptors at the membrane [118]. NETO2 is an accessory protein of KARs involved in the modulation of KAR functional properties (see Section 4.3 for details). Furthermore, GRIP is known to directly bind kinesin motor proteins [119], suggesting that GRIP binding to GluK1 or GluK2 may be involved in the transport and targeting of KARs.

**Table 2:** KAR-interacting proteins.

Interacting protein	Interacting domain	KAR subunit	Target domain/motif	Regulation of	Reference
**PDZ proteins**					
PSD-95	ND	GluK1b/c	-ETVA	ND	[79]
	PDZ1	GluK2	-ETMA	Desensitization	[104]
				and clustering	[104]
	GK	GluK5	ND	Clustering	[104]
	SH3	GluK5	914 P**P**GGPR**P** 920	Clustering	[104]
	SH3	GluK5	962 **P**PRPRPG**P** 969	Clustering	[104]
SAP-97	PDZ1	GluK2	-ETMA	Clustering	[104,115]
SAP-102	ND	GluK2	ND	Clustering	[104]
	ND	GluK5	ND	Clustering	[104]
PICK1	PDZ	GluK1b/c	-ETVA	Membrane	[79]
				anchoring	
	ND	GluK2	CTD	ND	[79]
GRIP	PDZ4-5	GluK1b/c	-ETVA	Membrane	[79]
				anchoring	
	PDZ4-5	GluK2	CTD	ND	[79]
Syntenin	PDZ2	GluK1b/c	-ETVA	ND	[79]
	ND	GluK2	CTD	ND	[79]
**Cytoskeleton**					
Cadherins	ND	GluK2	ND	Localization	[136]
				and trafficking	
*β*-catenin	ND	GluK2	CTD (indirect)	Membrane	[136]
				dynamics	
	ND	GluK5	ND	ND	[136]
p120 catenin	ND	GluK2	ND	ND	[136]
Velis	ND	GluK2	ND	ND	[136]
4.1N	ND	GluK1/2	MPD	Trafficking	[137]
Profilin II	ND	GluK2b	862 PP 863	Trafficking	[32,33]
Spectrin	ND	GluK2a	CTD	ND	[32]
**BTB/Kelch proteins**					
KRIP6	BTB/POZ	GluK2a	residues 842-899	Gating	[138]
Actinfilin	ND	GluK1	ND	ND	[139]
	Kelch repeats	GluK2	CTD	Degradation	[139]
**Kinases**					
CASK	ND	GluK2	-ETMA	ND	[136]
**Phosphatases**					
Calcineurin	ND	GluK2b	CTD	Function	[32,91]
**CUB proteins**					
NETO1	ND	GluK2/3/5	ND	Trafficking	[128,133]
	CUB2	GluK2/3/5	ND	Interaction	[124,128]
	ND	GluK1-5	ND	Function	[124,130]
	348 **RKK** 350	GluK2	ND	Rectification	[129]
NETO2	ND	GluK1/2	ND	Trafficking	[118,126]
	CUB2	GluK2/3/5	ND	Interaction	[124,128]
	LDLa	GluK1/2/5	ND	Function	[125,127,131]
	347 **RKK** 349	GluK2	ND	Rectification	[129]
**Other proteins**					
COPI	ND	GluK5	862 RRRRR 866	Trafficking	[35]
14-3-3	ND	GluK2a/5	ND	Trafficking	[32,35]
SNAP-25	ND	GluK5	CTD	Trafficking	[81]
Calmodulin	ND	GluK2	CTD	ND	[32]
Contactin	ND	GluK2a	CTD	ND	[32]
Dynamin-1	ND	GluK2a	CTD	ND	[32]
Dynamitin	ND	GluK2a	CTD	ND	[32]
NSF	ND	GluK2b	CTD	ND	[32]
VILIP1	ND	GluK2b	CTD	ND	[32]
VILIP3	ND	GluK2b	CTD	ND	[32]
Ubc9	ND	GluK2a	885VKTE888	Endocytosis	[96]
PIAS3	ND	GluK2a	885VKTE888	Endocytosis	[96]

KAR: Kainate receptor; ND: not determined; CTD: C-terminal domain; MPD: membrane-proximal domain.

### 4.3. CUB Domain-Mediated Interactions with KARs

Recently, two transmembrane proteins have been identified as accessory proteins of KARs and were shown to impact functional properties of both recombinant and native KARs. NETO1 and NETO2 (neuropilin and tolloid-like 1 and 2) proteins carry two extracellularly located CUB domains (complement C1r/C1s, Uegf, Bmp1), followed by a an extracellular juxtamembrane low-density lipoprotein class a (LDLa) domain and a transmembrane domain. Furthermore, NETO1 possesses a class 1 PDZ ligand domain and NETO2 a putative class II PDZ ligand at their respective C-termini [118,120]. Transcripts of both proteins are found in the brain and the retina [121,122,123]. Although NETO1 and NETO2 are both widely expressed in the brain, NETO1 is predominantly present in the hippocampus and almost completely absent from the cerebellum, whereas NETO2 shows modest expression in the hippocampus, but robust expression in the cerebellum [123,124].

Zhang *et al*. were the first to demonstrate that NETO2 interacts with KARs using a proteomic approach. Subsequently, NETO1 and NETO2 have been shown to interact with multiple other homomeric and heteromeric KARs in heterologous systems and in neurons (Table 2), thereby influencing pharmacological and gating properties, probably in a subunit-dependent manner [124,125,126,127,128,129,130,131]. While the general interaction of homomeric GluK2 receptors with NETO proteins is primarily established via the second CUB domain [128], the functional modulation of GluK2 appears to be mediated by the LDLa domain [127]. In support of these findings, modulation of KAR desensitization is diminished when the LDLa domain is mutated. Surprisingly, the effects of NETO proteins on rectification characteristics are not mediated via this domain. Instead, a series of three positively charged residues (RKK) near the intracellular side of the transmembrane domains of NETO1 and NETO2 appear to be responsible for mediating changes in rectification behavior [129].

Another prominent feature of many accessory proteins, for example TARPs (transmembrane AMPA receptor regulatory proteins), which interact specifically with AMPA receptors [132], is the regulation of receptor surface expression. Conflicting data have been published as to whether NETO proteins are involved in trafficking and synaptic localization of KARs [118,120,124,126,127,128,133].

For GluK1 it has been reported that NETO2 promotes surface expression and synaptic localization of the receptors in transfected hippocampal neurons, while NETO1 seems to lack this feature and rather reduces neuronal GluK1 plasma membrane localization [126]. Additionally, Tang *et al*. provide evidence for a reduced abundance of GluK2-containing KARs in the PSD of cerebella from NETO2 knock-out mice, while the overall GluK2 expression remains unchanged [118]. However, NETO2 does not affect the surface expression of GluK2 in the heterologous *X. laevis* oocyte system. Vice versa, at least in *Xenopus* oocytes, NETO2 plasma membrane expression appears to be promoted by association with GluK2. This is reflected in a strong decrease in NETO2 plasma membrane expression in the cerebella from GluK2 knock-out mice [127]. Furthermore, NETO2 interacts with the scaffolding protein GRIP, suggesting NETO2 to play a role in promoting or stabilizing GluK2/GRIP interactions and ultimately stabilizing receptor complexes at synapses [118].

Regarding NETO1, it has been reported that NETO1 knock-out mice and NETO1/2 knock-out mice exhibit a drastically reduced abundance of GluK2- and GluK5-containing KARs in the PSDs of hippocampal neurons, while a lack of NETO2 has no obvious impact on receptor abundance. Importantly, the overall abundance of hippocampal GluK2 and GluK5 remains unchanged [128]. In agreement with these results, Wyeth *et al*. confirm by postembedding immunoelectron microscopy that GluK2/3 immunogold labeling is reduced in mossy fiber-CA3 PSDs in the hippocampi of NETO1 knock-out mice and NETO1/2 knock-out mice [133]. By contrast, Straub *et al*. report that neither GluK2/3 nor GluK5 surface expression in acute hippocampal slices nor their localization in the hippocampus are affected in NETO1 knock-out mice [124].

However, it cannot be excluded that these apparent discrepancies regarding possible implications of NETO proteins in synaptic localization of KARs might be caused by different genetic backgrounds of the mice knock-out lines used in these experiments. Another possibility is that NETO-mediated trafficking is highly cell type-, receptor subunit- and NETO isoform-dependent, therefore explaining some of the conflicting data. Nevertheless, EPSCKARs have been shown to be present even in NETO1/2 knock-out mice [124,128], indicating that NETO proteins are not required for the targeting of KARs to synapses, but might rather be implicated in clustering and functional modulation of KARs.

Furthermore, Ng *et al*. report that NETO1 interacts with GluN2A/B and is implicated in the maintenance of GluN2A-containing NMDA receptors within the PSD of hippocampal neurons [120]. Furthermore, NETO1 associates with the NMDA receptor/amyloid precursor protein (APP) complex and is therefore believed to be involved in regulation of NMDA receptor surface expression [134]. Nevertheless, the precise mechanism how NETO1 functions in the APP/NMDA receptor trafficking complex remains elusive [134]. Whether NETO1 is implicated in the delivery of NMDA receptors to the synapse or stabilizing the synaptic abundance of NMDA receptors is still a matter of discussion (see also [124,133]).

Another recent finding reports NETO2 to interact with KCC2, a neuron-specific K^+^-Cl*^−^* cotransporter implicated in Cl*^−^* homeostasis and fast inhibitory synaptic transmission, therefore demonstrating NETO2 to be a multifunctional protein regulating proteins in excitatory and inhibitory neurotransmission [135].

### 4.4. Interactions of KARs with Proteins of the BTB/Kelch Family

Another protein known to interact with KARs is KRIP6 (kainate receptor interacting protein for GluR6). KRIP6 belongs to the BTB/Kelch family (BTB: broad-complex, tram-track, and bric-a-brac; Kelch: named after a sixfold tandem element in the sequence of the *D. melanogaster* Kelch ORF1 protein) and binds to the C-terminus of GluK2a (Table 2). This binding is probably established via the BTB/POZ (broad-complex, tram-track, and bric-a-brac/poxvirus and zinc finger) domain within the N-terminus of KRIP6. KRIP6 alters functional properties of GluK2a by reducing peak current amplitude and steady-state desensitization (ratio of steady-state to peak current after pre-exposure of channels to non-saturating agonist concentrations) in heterologous expression systems. Its overexpression in hippocampal neurons reduces KAR-mediated responses. However, KRIP6 interaction with GluK2a does not affect plasma membrane expression of the receptor [138]. Additionally, KRIP6 interacts with PICK1 [140], which associates with KARs via its PDZ domain [79]. Laezza *et al*. show that PICK1, in marked contrast to KRIP6, increases peak current and relative desensitization of GluK2 in heterologous expression systems. Furthermore, simultaneous expression of KRIP6 and PICK1 cancels out their opposing effects on GluK2 function. Additionally, it has been shown that PICK1 and KRIP6 co-cluster even in the absence of GluK2, suggesting a mechanism to regulate physiological properties of KARs by two proteins with opposite regulatory functions, in which KRIP6 might serve as a dominant negative regulator reducing PICK1/GluK2 association [140].

In addition, the BTB/Kelch protein actinfilin binds to GluK2 (Table 2) and with lower affinity to GluK1 and is implicated in targeting of GluK2 for degradation [139]. Actinfilin attaches to the C-terminus of GluK2 via its Kelch repeats. Furthermore, actinfilin interacts via its BTB/POZ domain with Cul3 (Cullin 3) [139], which functions as a scaffold protein for the Cul3-based E3 ubiquitin-ligase complex [141,142,143], suggesting that actinfilin might be implicated in degradation of GluK2-containing receptors. Indeed, GluK2 ubiquitination is increased in the presence of actinfilin and accompanied by a decrease in plasma membrane abundance of the receptor in heterologous expression systems. In support of these findings, GluK2 levels are increased in brain synaptosomes of heterozygous Cul3^+^*^/^^−^* mice, while ubiquitination is reduced. Furthermore, RNAi of actinfilin in hippocampal neurons increases GluK2 surface expression in dendritic spines, suggesting the actinfilin-Cul3-mediated degradation of GluK2 to be an important mechanism for regulating synaptic GluK2 levels [139].

### 4.5. Interactions with Cell Adhesion Proteins and the Cytoskeleton

Stabilization of KARs at synaptic junctions is further regulated by interactions with proteins involved in cell adhesion processes (Table 2). Coussen *et al*. provide evidence for GluK2-containing KAR interactions with cadherins, *β*-catenin, p120 catenin, CASK (calcium/calmodulin-dependent serine protein kinase 3), and Velis [136], therefore linking GluK2 to proteins responsible for associating cell adhesion molecules with PSD proteins and neurotransmitter receptors [144,145,146]. However, binding of *β*-catenin to the CTD of GluK2 is established indirectly. Nevertheless, the reorganization of cadherin/catenin complexes during formation of synaptic junctions is paralleled by a redistribution of GluK2, therefore suggesting a role of cadherin/catenin complexes in targeting or stabilizing GluK2 at synaptic junctions. Thus, changes in adhesiveness may provide a mechanism to regulate synaptic properties [136].

Additionally, neuroligin interacts with PSD-95, SAP-102, and PICK1 [147]. Neuroligin is located in the postsynaptic plasma membrane and is implicated in regulating the postsynaptic abundance of NMDA receptors [148]. Since neuroligin interacts with proteins also known to interact with GluK1 and GluK2 [79,104], it is tempting to speculate that neuroligin might play a role in controlling postsynaptic KAR abundance as well.

Furthermore, GluK1 and GluK2 interact with the spectrin-actin binding scaffolding protein 4.1N via a membrane-proximal domain of their CTDs [137]. 4.1N proteins possess a spectrin-actin binding domain, allowing them to function as cytoskeletal adaptor proteins [149]. The interaction of 4.1N with GluK1/2 is regulated by palmitoylation and phosphorylation of the KAR subunits. Distal palmitoylation of the CTD of GluK2a promotes association of 4.1N, resulting in an increased membrane stability of GluK2 due to a reduced constitutive internalization of this receptor. This effect is opposed by PKC-dependent phosphorylation. Phosphorylation of the membrane-proximal residue S846 has the strongest effect on 4.1N association with GluK2a. In summary, 4.1N association with KARs and its regulation by palmitoylation and phosphorylation might constitute a critical mechanism for the fine-tuning of synaptic transmission [137].

## 5. Conclusions

Considerable progress in understanding the function of KA receptors (KARs) and their role during synaptic plasticity has been made during the past years. However, many aspects of the dynamic regulation of their biosynthesis, synaptic targeting, and functional modulation still remain elusive. Intracellular retention of KARs appears to involve several key quality control mechanisms that cannot be explained solely by the existence of ER retention signals. Apparently, structural rearrangements, agonist binding, and a complex interplay between the biosynthetic pathways of ER-resident proteins and interacting cytosolic proteins determine the efficiency of trafficking of KARs. The knowledge about endocytic mechanisms controlling the surface expression also progressed significantly. However, apparent discrepancies regarding the induction and expression of KAR-mediated LTD suggest that one or several key players involved in this process may still await discovery. Furthermore, the exact mechanisms underlying polarized trafficking of KARs still need to be elaborated. One of the most promising directions to further elucidate the role of KARs in synaptic plasticity will therefore be the better understanding of the function of KAR-interacting proteins. For example, NETO proteins have just recently been recognized to significantly impact the function and possibly the trafficking of KARs. It would be of great interest to further characterize this interaction and elucidate how the NETO-dependent modulation of KARs is regulated. Until now, it is unclear whether NETO proteins are involved in KAR trafficking, how this possible regulation could be established, and whether NETO proteins themselves are post-translationally modified. Furthermore, the stoichiometry of NETO/KAR complexes has not yet been determined, and whether changes in this stoichiometry may impact KAR function or abundance can only be speculated. Interestingly, NETO proteins have been shown to interact with proteins known to influence NMDA receptor trafficking and synaptic abundance. Thus, understanding the impact and regulation of these complex mutual interactions between neurotransmitter receptors and associated proteins would be of great fundamental interest. In addition, understanding these interactions at the molecular level possibly would allow to improve the therapeutic possibilities to treat putative KAR-related diseases, such as ischemia, persistent pain perception, and schizophrenia (see [12] for a review on KARs and their putative connection to certain diseases).

## Definitions

AMPA: *α*-amino-3-hydroxy-5-methylisoxazole- 4-propionic acid
APP: amyloid precursor protein
BTB: broad-complex, tram-track, and bric-a-brac
BTB/POZ: broad-complex, tram-track, and bric-a-brac/poxvirus and zinc finger
CaMK: calmodulin-dependent kinase
CASK: (calcium/calmodulin-dependent serine protein kinase
CNS: central nervous system
COPI: coatomer protein complex I
COS: CV-1 (simian) in origin, carrying SV40 genetic material
CTD: C-terminal domain
CUB: complement C1r/C1s, Uegf, Bmp1
Cul3: Cullin 3
DG: dorsal root ganglia
EndoH: endoglycosidase H
EPSC: excitatory postsynaptic current
ER: endoplasmic reticulum
GABA: γ-aminobutyric acid
GK: guanylate kinase
GRIP: glutamate receptor interacting protein 1
iGluR: ionotropic glutamate receptor
JNK: c-Jun N-terminal kinase
KA: kainate
KAR: kainate receptor
KCC2: potassium chloride cotransporter 2
KRIP6: kainate receptor-interacting protein for GluR6
LBD: ligand binding domain
LDLa: low-density lipoprotein class a
LTD: long-term depression
LTP: long-term potentiation
MAGUK: membrane-associated guanylate kinase
mGluR: metabotropic glutamate receptor
MLK: mixed-lineage kinase
Nbea: neurobeachin
NETO: neuropilin and tolloid-like
NMDA: N-methyl-D-aspartate
NTD: N-terminal domain
PDZ: post synaptic density protein (PSD-95), Drosophila disc large tumor suppressor (Dlg1), and zonula occludens-1 protein (zo-1)
PICK1: protein interacting with PRKCA 1
PKA: cAMP-dependent protein kinase
PKC: protein kinase C
PSD: postsynaptic density
RNA: ribonucleic acid
RNAi: RNA interference
SAP: synapse-associated protein
SENP: sentrin-specific protease
SH3: Src homology 3
SNAP: synaptosomal-associated protein
SUMO: small ubiquitin-related modifier
TMD: transmembrane domain
